# mTORC2 disruption reprograms nutrient-driven behavioral adaptations in *C. elegans*

**DOI:** 10.17912/micropub.biology.001794

**Published:** 2025-09-11

**Authors:** Simran Motwani, Somya Bhandari, Arnab Mukhopadhyay

**Affiliations:** 1 Molecular Aging Laboratory, National Institute of Immunology, New Delhi, Delhi, India

## Abstract

Animals adapt their foraging behavior based on the quality and availability of food in the environment. Here, we examine how the disruption of mTORC2 affects such adaptations in *Caenorhabditis elegans* . Specifically, we characterize the food preference and dwelling behavior of the mutant of the *rict-1* gene, which codes for the core component of mTORC2, across two diets of differing nutritional value. Our findings show that *rict-1* mutants display altered behavioral responses shaped by prior dietary experience, indicating impaired dietary memory. This study reveals a critical role for mTORC2 signaling in integrating past nutritional states with current foraging decisions, providing insight into how metabolic pathways regulate context-dependent feeding behavior in *C. elegans* .

**Figure 1. Loss of rict-1 reshapes diet-driven behavioral preferences in C. elegans:**
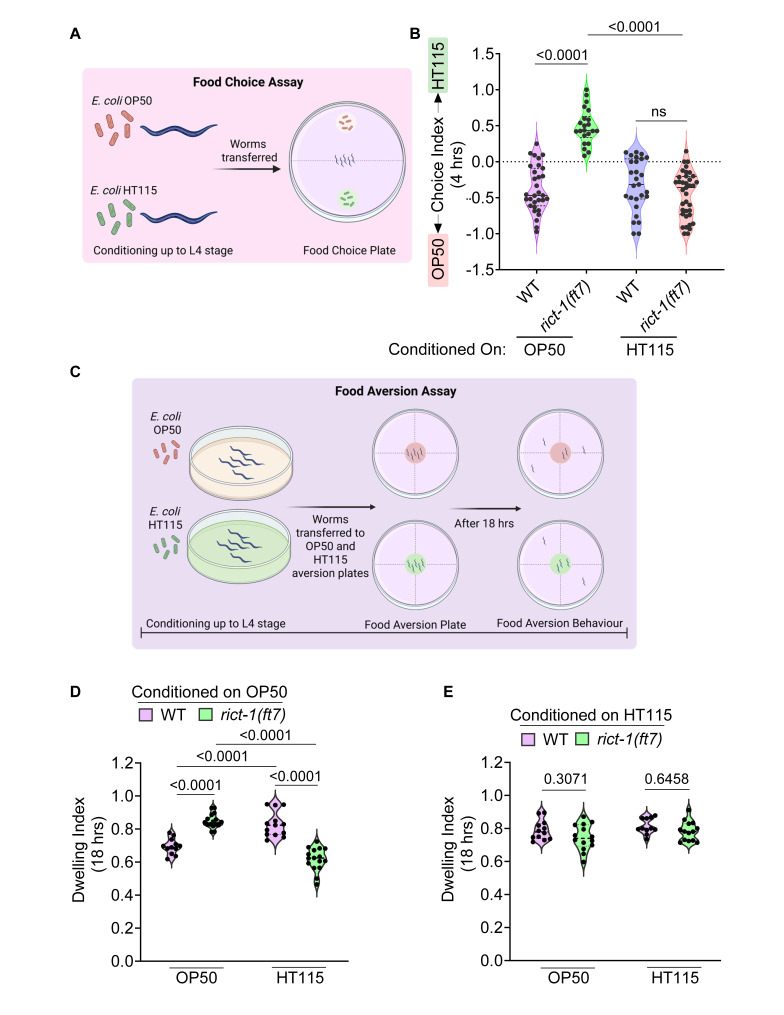
**Figure 1:** Loss of *rict-1* reshapes diet-driven behavioral preferences in *C. elegans* . **(A)** Illustration of the experimental setup used to assess food choice behavior. **(B)** The *rict-1* mutant conditioned on the OP50 diet displayed a preference for HT115 , and those conditioned on HT115 favored OP50 . In contrast, wild-type animals, regardless of dietary conditioning, consistently chose OP50 . P-value determined using Kruskal-Wallis test with Dunn's multiple comparisons test. Each dot represents a worm. The experiment has been performed thrice. **(C)** Diagram summarizing the food aversion assay. **(D)** When conditioned on OP50 , *rict-1* mutants spent more time dwelling on OP50 as compared to HT115 . Wild-type animals showed the opposite pattern under the same conditions. P-value determined using Ordinary Two-way ANOVA with Tukey's multiple comparisons test. Each dot represents a biological replicate. **(E)** Upon conditioning with HT115 , *rict-1* mutants no longer exhibited strong dietary bias in their dwelling behavior and responded similarly to both bacterial lawns, mirroring wild-type responses. P-value determined using Ordinary Two-way ANOVA with Tukey's multiple comparisons test. Each dot represents a biological replicate. The schematics were created using BioRender. The source data for panels B, D and E can be found in the extended data section.

## Description

Early life nutrition can profoundly shape adult feeding behavior and metabolic consequences (Vickers, 2014; Waterland & Michels, 2007), but the molecular basis of these adaptive responses is less understood. We sought to elucidate the function of the Mechanistic Target of Rapamycin Complex 2 (mTORC2) by mutating an essential core component, RICT-1 , required for the stability of the complex (Aimbetov et al., 2012), in the modulation of dietary experience-dependent behavior in *Caenorhabditis elegans* . mTORC2's role in metabolism and stress response is well described (Sciarretta et al., 2015; Szwed et al., 2021), but its role in the modulation of behavior according to previous dietary experiences is less known. To investigate this, we utilized the null allele, *rict-1 ( ft7 )* , and compared its behavior to wild-type worms. Animals were conditioned from the L1 larval stage onwards on either *Escherichia coli* OP50 (a ‘low-quality' diet) or *E. coli* HT115 (a ‘high-quality' diet) (Zecic et al., 2019). Behavioral assays performed at the L4 stage showed how early dietary exposure influenced later food choice and food-associated dwelling behavior.

In the food choice assay, worms were positioned in the center of assay plates with equidistant spots of OP50 and HT115 ( **Figure 1A** ), and their position after four hours was imaged using WormLab (MBF Bioscience, USA) and quantified. Wild-type worms displayed a dietary preference biased toward OP50 , whether conditioned on OP50 or HT115 ( **Figure 1B** ). On the contrary, *rict-1 ( ft7 )* animals showed a dramatic experience-dependent food choice. Conditioned on OP50 , mutants chose HT115 preferentially, while when conditioned on HT115 , they chose OP50 ( **Figure 1B** ). This bidirectional switch in food choice suggests that *rict-1* mutants may retain memory of early dietary experience that determines choice in adulthood, implicating mTORC2 in modulating dietary memory.

In the dwelling behavior assay, worms were plated on a single patch of food ( HT115 or OP50 ) ( **Figure 1C** ) and their positions were scored after 18 hours. Wild-type animals conditioned on OP50 dwelled longer on HT115 than on OP50 , implying an adaptive behavior favoring an overall better diet ( **Figure 1D** ). In contrast, OP50-conditioned *rict-1 ( ft7 )* animals exhibited greater dwelling on OP50 , again, indicating reversal of adaptive food-related behavior ( **Figure 1D** ). Notably, HT115-conditioned wild-type or *rict-1 ( ft7 )* animals exhibited similar dwelling behavior on either food source, indicating that mTORC2-mediated modulation is more important after exposure to nutritionally poor environments ( **Figure 1E** ).

Together, these results define RICT-1 /mTORC2 as a critical modulator of experience-dependent behavioral plasticity tied to early nutritional status. In addition to its known functions in metabolism (Soukas et al., 2009; Szwed et al., 2021), mTORC2 is here revealed as a central molecular mediator linking early nutrition to subsequent behavioral adaptation. Strengths of this work include the utilization of a *rict-1 ( ft7 )* mutant, which is widely accepted as a mTORC2 loss-of-function allele (Gatsi et al., 2014; Ruf et al., 2013; Sakai et al., 2017), the combination of two behavioral paradigms (choice and dwelling), and the controlled environmental conditions. However, we note that employing independent alleles of *rict-1* or rescue experiments will be necessary to confirm our conclusions. Also, the specific neuronal circuits and downstream effectors of mTORC2's behavioral effect were not determined and need to be explored further. Moreover, the assays employed only two bacterial diets, and the inclusion of a wider variety of diets would serve to establish whether the reversal is specific to nutrient quality or might be generalized across other types of dietary experience.

The idea that early-life dietary environments have the ability to influence long-term behavioral and metabolic consequences is becoming more widely accepted in human health research (Vickers, 2014). In humans, early life exposure to specific types of diets, for example, high-fat, high-sugar diets, has been associated with altered taste preferences, overeating, and risk of metabolic syndromes like obesity and diabetes (Zhou et al., 2020).

In summary, our research uncovers RICT-1 /mTORC2 as a vital mediator of adult food preference and dwelling behavior brought about by early dietary experience in *C. elegans* , presenting a model to expand the molecular foundation of the developmental origins of disease and health.

## Methods

**Strains and Conditioning**

Wild-type ( N2 Bristol) and *rict-1 ( ft7 )* worms were cultured on the OP50 diet at 20°C. For conditioning, synchronized L1 larvae (by bleaching) were transferred to NGM plates seeded with tetracycline-resistant *E. coli* OP50 or *E. coli* HT115 . Worms were cultured on the diets until the L4 stage.

**Bacterial growth**

Bacteria stored in glycerol stocks were streaked onto Luria-Bertani (LB) plates containing tetracycline (12.5 µg/ml) and then allowed to incubate at 37°C for 16 hours until distinct single colonies became visible. A single colony was then used to inoculate LB broth and grown overnight at 37°C for the primary culture. The secondary cultures were initiated with a 1/100th volume of the primary culture and left to incubate at 37°C for 2 hours ( HT115 ) or 2.5 hours ( OP50 ) at 165 rpm. The bacterial pellets were then resuspended in 1/10th volume of 1XM9 buffer. Approximately 500 µl of bacterial suspension was spread on NGM plates and left to dry for 1 day.

**Food Choice Assay**

Primary cultures of HT115 and OP50 bacteria were grown overnight, then sub-cultured in fresh LB broth for 2 hours ( HT115 ) or 2.5 hours ( OP50 ) at 37°C with shaking (165 rpm) till it reached an OD between 0.6-0.8. Bacterial pellets were resuspended in 1/10 volume of 1× M9 buffer. Binary-choice assay plates were prepared by spotting 20 µl of each bacterial suspension at opposing positions on 60 mm NGM plates, followed by 16-hour incubation at 20°C. Synchronized L4-stage worms, pre-conditioned on specific diets, were washed three times in 1× M9 buffer to remove residual bacteria. Approximately 30–50 worms were transferred to the center of the assay plate. After a 4-hour incubation at 20°C, worms were scored based on their location relative to the HT115 or OP50 food patches. The preference index (I) was calculated as I = (NH - NO)/(Total number of worms), where NH is the number of worms on the HT115 diet, and NO is the number of worms on the OP50 diet.

**Food Dwelling Assay**

Bacterial cultures ( HT115 or OP50 ) were prepared as above. For dwelling assays, 60 µl of bacterial suspension was spotted at the center on a 60 mm NGM plate and incubated for 16 hours at 20°C. Conditioned L4-stage worms were washed and transferred to the center of the food spot. After 18 hours, worms were categorized as “on-food” (within the bacterial lawn) or “off-food” (outside the lawn). The dwelling index was the percentage of worms still on the food spot after the exposure period compared to the total number of worms.

**Statistical Analysis**

Statistical analysis was done using GraphPad Prism 10. P-values less than 0.05 were taken as statistically significant.

## Reagents

**Table:** 

**Strain Used**	**Genotype**	**Source**	**Description**
N2	Wild-type	CGC	Standard laboratory strain
*rict-1 ( ft7 ) *	*rict-1* null mutant	CGC	mTORC2 signaling-defective mutant

## Data Availability

Description: Source data file. Resource Type: Dataset. DOI: https://doi.org/10.22002/7t7x5-cg661
